# An erosion control and soil conservation method for agrarian uses based on determining the erosion threshold

**DOI:** 10.1016/j.mex.2018.07.007

**Published:** 2018-07-09

**Authors:** Rafael Blanco-Sepúlveda

**Affiliations:** Geographic Analysis Research Group, Geography Dept., University of Málaga, 29071 Málaga, Spain

**Keywords:** Soil conservation method based on determining the erosion threshold, Soil erosion, Visual indicators, Erodibility factors, Environmental sustainability

## Abstract

We present a practical method to control erosion and soil conservation for agrarian activity. The method consists of four steps: (1) analyse the erosive state (as a percentage of the area affected) and soil losses (in m^3^ ha^−1^) by means of visual indicators (physical features on the soil surface that have been formed by erosion) and analyse environmental factors (as possible erodibility factors); (2) determine the factors of erodibility; (3) calculate the erosion threshold (value of the erodibility factor beyond which effective control of erosion is achieved), by statistical analysis of the databases collected in the field; (4) verify this result by applying a qualitative assessment of erosive intensity, using visual indicators. The erosion threshold is of particular interest for practical purposes because it can be used to improve the planning of agricultural activity, from the standpoint of soil conservation, thus promoting sustainable land use.

This study makes the following main contributions to knowledge in this field:

• It presents a new method to address erosion control, based on determining the erosion threshold.
• Appropriate environmental and management conditions for effective erosion control are identified.
• The method is simple to apply, which facilitates its implementation.

## Graphical abstract

**Figure:**
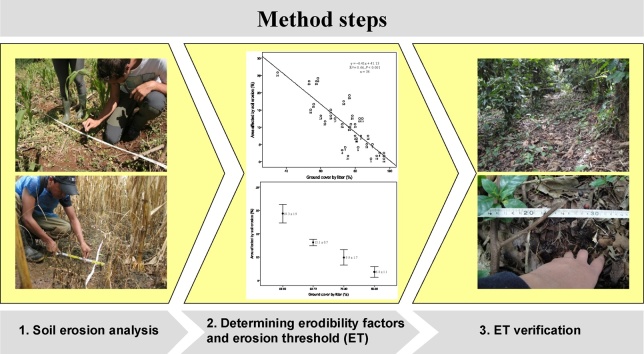

**Specifications Table**

**Table:** 

*Subject area*:	*Environmental Science*
*Method name*:	Soil conservation method based on determining the erosion threshold

## Background

This paper presents a method of soil erosion analysis for determining erodibility factors by means of visual indicators and, ultimately, the erosion threshold (ET) to achieve erosion control and soil conservation.

Field methods to study erosion at the plot scale differ in precision, equipment and costs. The most precise techniques, which are usually the most expensive, do not always best match the research aims [1]. Precise measurements obtained at one site may not be representative of the entire study area [2]. This situation can lead to problems of representativeness in study areas featuring large temporal and spatial variations of erosion. A further problem is that of the difficulty often encountered in applying these methods in remote and/or inaccessible areas, or in areas with heterogeneous environmental characteristics, with different types of soil and biomass management for crops and pastures.

Accordingly, other approaches have been used, based on the assessment of soil erosion using visual indicators to attenuate these drawbacks. Such methods present the following advantages: (a) they are low cost, not requiring infrastructure or specialised equipment; (b) they are simple and fast to apply because they are based on assessing erosion from visual indicators; (c) therefore, a large number of plots can be sampled, thus providing a sufficient volume of data to ensure that the results obtained are representative of the study area.

The erosion study methods presented in this paper improve upon traditional approaches by quantitatively assessing soil erosion, using sampling methods. Furthermore, the use of random sampling methods ensures that the results obtained are representative of the study plots. While traditional methods have exclusively used visual indicators of erosion, we also take into account surface processes that affect the soil (erosive impacts, mechanical impacts by trampling or the use of tools, deposit processes and soil protected from erosion, compaction and mechanical impact caused by animal transit). With this approach, the evaluation of the erosive state is complemented by an assessment of the processes that affect the soil and that can influence erosion.

With respect to erosion control and soil conservation, the erosion threshold (ET), defined as “the value (of the erodibility factor) beyond which effective control of erosion is achieved” [3], can be considered to belong to the set of methods that take the sustainability of agrarian activities into account. Its main forerunner was the approach based on analysing the tolerable level of soil erosion (T), defined as “the maximum rate of soil erosion that will permit a high level of crop productivity to be sustained economically and indefinitely” [4]. T, although it reflects a markedly economic approach to the question, also has the aim of physically preserving the soil, as its conservation is the basis for maintaining soil fertility in the long term. This aspect is made explicit in certain definitions of the concept that incorporate the idea that a balance between soil losses and gains must be maintained [5]. Therefore, ET is clearly complementary to the goal represented by T, as the approach designed to establish ET seeks to achieve the environmental and management conditions needed for effective control of erosion.

## Method details

The method includes the following steps:

1 Analysis of soil erosion and environmental factors (as possible erodibility factors)
2 Determination of erodibility factors.
3 Calculation of the erosion threshold.
4 Verification of the erosion threshold.

### Analysis of soil erosion and environmental factors (as possible erodibility factors)

In the analysis of soil erosion, the following aspects must be calculated:

(a) the state of soil erosion, observing the surface processes affecting the soil, including all types of erosion (splash, sheet, rills and gullies), expressing the results as a percentage of the surface area affected.
(b) the soil loss by erosion in rills and gullies, expressing the results as a volume (m^3^ ha^−1^).

The erosive state is analysed using an adapted version of the presence/absence test of visual indicators and their number and size [6,7]. The visual indicators are differentiated using codes to streamline data collection during field sampling (Table 1, Table 2). The index (capital letter) indicates the type of process that affects the soil, and the subindex provides complementary information, such as the existence of erosion, the erosion type, the type of protective vegetal cover, etc. Types E, P, T, D and C correspond to cases of erosion. The analysis distinguishes between erosion directly affecting unaltered soil (E), erosion affecting soil previously disturbed by agricultural practices with implements, mainly in ploughing the ground to prepare it for planting and to remove residues (P), the erosion of land affected by human or animal trampling (T), erosion affecting diffuse accumulations of materials on the slope, deposited in previous erosive events (D) and the erosion affecting soil that has been compacted by cattle trampling (C). This differentiation is performed in order to analyse and determine the influence of these activities (P, T and C) and processes (E and D) on the dynamics of erosion.

**Table 1:** Visual indicators of surface processes affecting the soil for different crops.

**E. Soil erosion by water**
1. (Ei) Splash erosion (raindrop impact)
2. (Es) Sheet erosion
3. (Er) Rill erosion
4. (Eg) Gully erosion

**P. Mechanical soil disturbance by tool** (machete, plough, others), **affected by erosion or erosion-susceptible**
1. (Pes) Soil disturbed by tool use and susceptible to erosion (but not visible at present)
2. (Pi/Ps/Pr/Pg) Soil disturbed by tool use and affected by splash erosion (raindrop impact)/by sheet erosion/by erosion in rills/by erosion in gullies

**T. Mechanical soil disturbance by trampling, affected by erosion or erosion-susceptible**
1. (Tes) Soil disturbed by trampling and susceptible to erosion (but not visible at present)
2. (Ti/Ts/Tr/Tg) Soil disturbed by trampling and affected by splash erosion (raindrop impact)/by sheet erosion/by erosion in rills/by erosion in gullies

**D. Soil deposition affected by erosion, or erosion-susceptible**
1. (Des) Soil deposition susceptible to erosion (but not visible at present)
2. (Di/Ds/Dr/Dg) Soil deposition affected by splash erosion (raindrop impact)/by sheet erosion/by erosion in rills/by erosion in gullies

**N. No erosion**
1. (Nw) No evidence of erosion beneath cover of weeds
2. (Nl) No evidence of erosion beneath litter layer
3. (Nf) No evidence of erosion in ploughing furrows with zero slope

**O. Others**
1. (Or) Stones
2. (Oa) Animals: tracks, faeces, carcasses, others.

**Table 2:** Visual indicators of surface processes affecting the soil for grazing use.

**E. Soil erosion by water**
1. (Ei) Splash erosion (raindrop impact)
2. (Es) Sheet erosion
3. (Er) Rill erosion
4. (Eg) Gully erosion

**C. Soil compaction by trampling (platy soil structure), affected by erosion or erosion-susceptible**
1. (Ces) Soil compacted and susceptible to erosion (but not visible at present)
2. (Ci/Cs/Cr/Cg) Soil compacted and affected by splash erosion (raindrop impact)/by sheet erosion/by erosion in rills/by erosion in gullies

**T. Mechanical soil disturbance by trampling in water-saturated soil (hoof slides, hoof imprints, subsidences in the cattle track), affected by erosion or erosion-susceptible**
1. (Tes) Soil disturbed by trampling and susceptible to erosion (but not visible at present)
2. (Ti/Ts/Tr/Tg) Soil disturbed by trampling and affected by splash erosion (raindrop impact)/by sheet erosion/by erosion in rills/by erosion in gullies

**N. No erosion**
1. (Nw) No evidence of erosion beneath cover of weeds
2. (Nl) No evidence of erosion beneath litter layer

**O. Others**
1. (Or) Stones
2. (Oa) Animals: faeces, carcasses, others.

Type N refers to soil that is not affected by erosion, and includes that which is protected by ground cover (weeds and litter layer) together with the presence of micro-reliefs with zero slopes, such as furrows caused by the plough that follow in parallel the contours of the slope (Nf). Finally, type O (other) incorporates all aspects of soil that are unrelated to erosion and which may be present on the soil surface: stones and evidence of animals (tracks, faeces, carcasses, etc.).

**Fig. 1:**
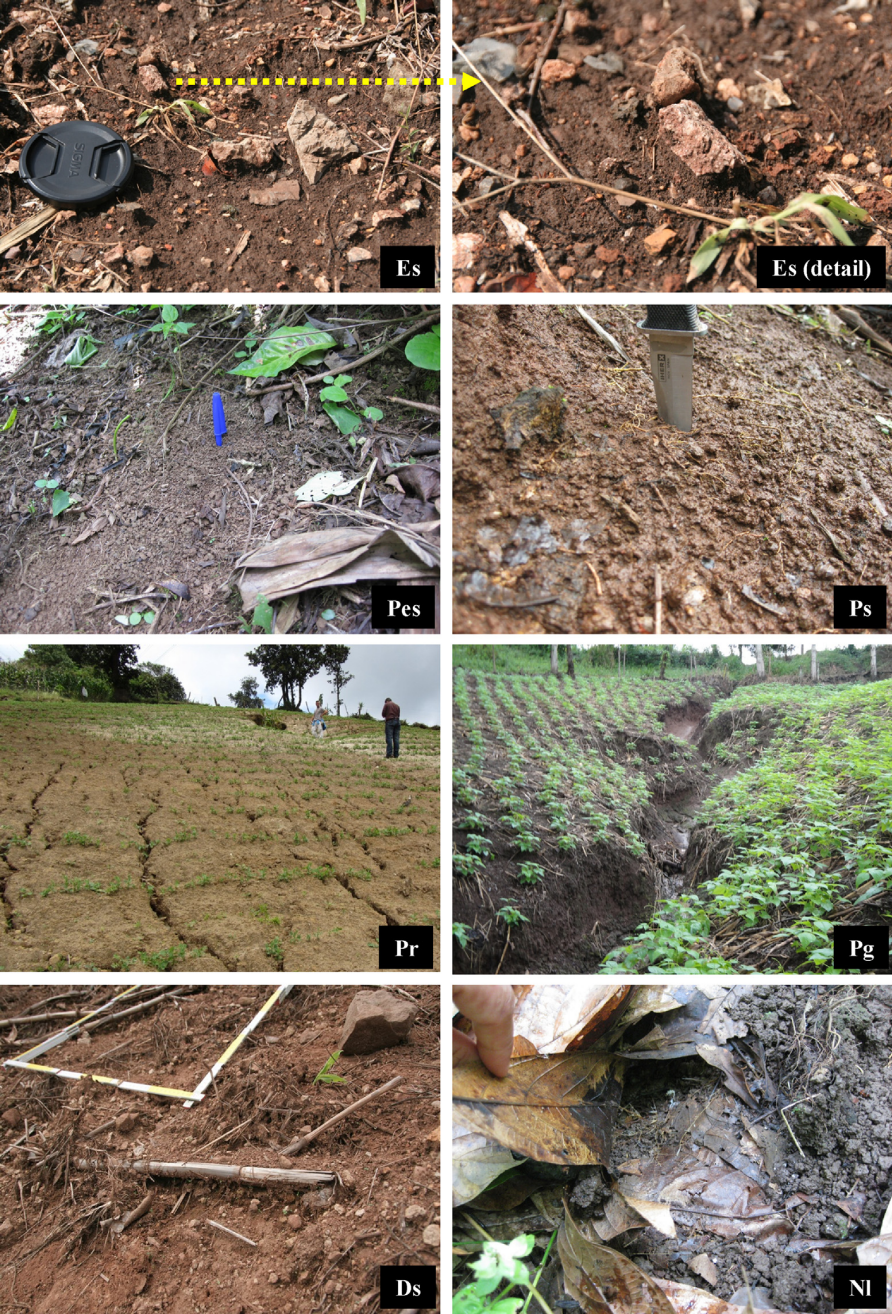
Visual indicators of surface processes affecting the soil for different crops. (Es) Sheet erosion in a maize crop. Numerous erosion pedestals can be seen (detail photo). (Pes) Soil disturbed by weed removal using a machete in a coffee crop. This action has destructured the soil to a depth of 2–3 cm, creating a granular-type structure (<1 cm) that is highly susceptible to erosion. (Ps) The soil type has evolved from Pes (the above case) to Ps (soil disturbed by weed removal and affected by sheet erosion). The granular structure has disappeared because the particles are completely bonded or have been washed away. (Pr) Soil disturbed by seeding using a hoe, and affected by rill erosion in a vetch crop. (Pg) Soil disturbed by seeding using a plough and affected by gully erosion in a bean crop. (Ds) Deposition of soil, favoured by the maize stalks remaining on the soil surface, affected by sheet erosion, in a maize crop. (Nl) Non-eroded soil beneath a cover of litter layer in a cacao crop. Under this cover, decomposing plant remains are visible, which indicates that the soil is not affected by erosion.

**Fig. 2:**
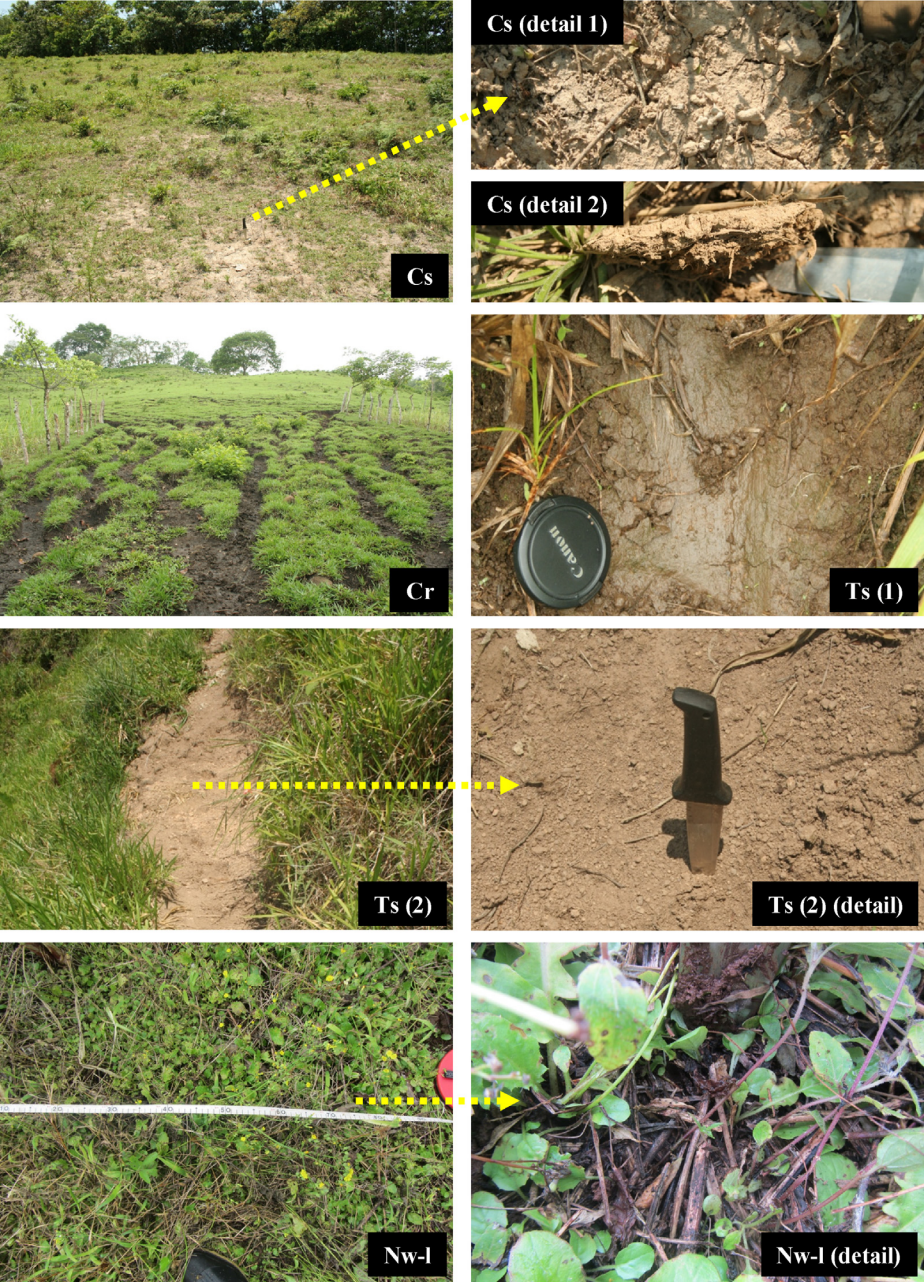
Visual indicators of surface processes affecting the soil in grazing use. (Cs) Soil compacted by trampling and affected by sheet erosion (detail 1). Platy soil structure can be seen (detail 2). (Cr) Soil compacted and affected by rill erosion. (Ts 1) Soil disturbed by hoof slide and affected by sheet erosion. (Ts 2) Soil disturbed by subsidence in the cattle track and affected by sheet erosion (detail photo). Subsidence is a common feature of cattle tracks (caused by the weight of the animals) and in pastures with extreme slopes when the soil becomes saturated. This process breaks up the soil structure and provokes a micro-slope in the track that facilitates erosion. (Nw-l) Non-eroded soil beneath a cover of weeds and litter. Under this cover, the soil is not affected by erosion (detail photo).

Of particular interest are activities P, T and C. The first, because ploughing and weeding with tools (different types of machetes) breaks up the original structure of the topsoil and makes it easier for surface runoff to carry away soil particles. Activities T and C are of interest because trampling by the transit of persons and animals alters the structure and physical properties of soil, such as porosity and infiltration capacity, which promotes erosion. Fig. 1, Fig. 2 illustrate the common visual indicators referred to in Table 1, Table 2.

In erosion analysis, soil losses are estimated by volumetric measurements of the rills and gullies [4]. The volume of soil lost is calculated assuming the cross-sections of these features have a geometric shape, whether semi-elliptical, semicircular, triangular or trapezoidal [6,8].

**Fig. 3:**
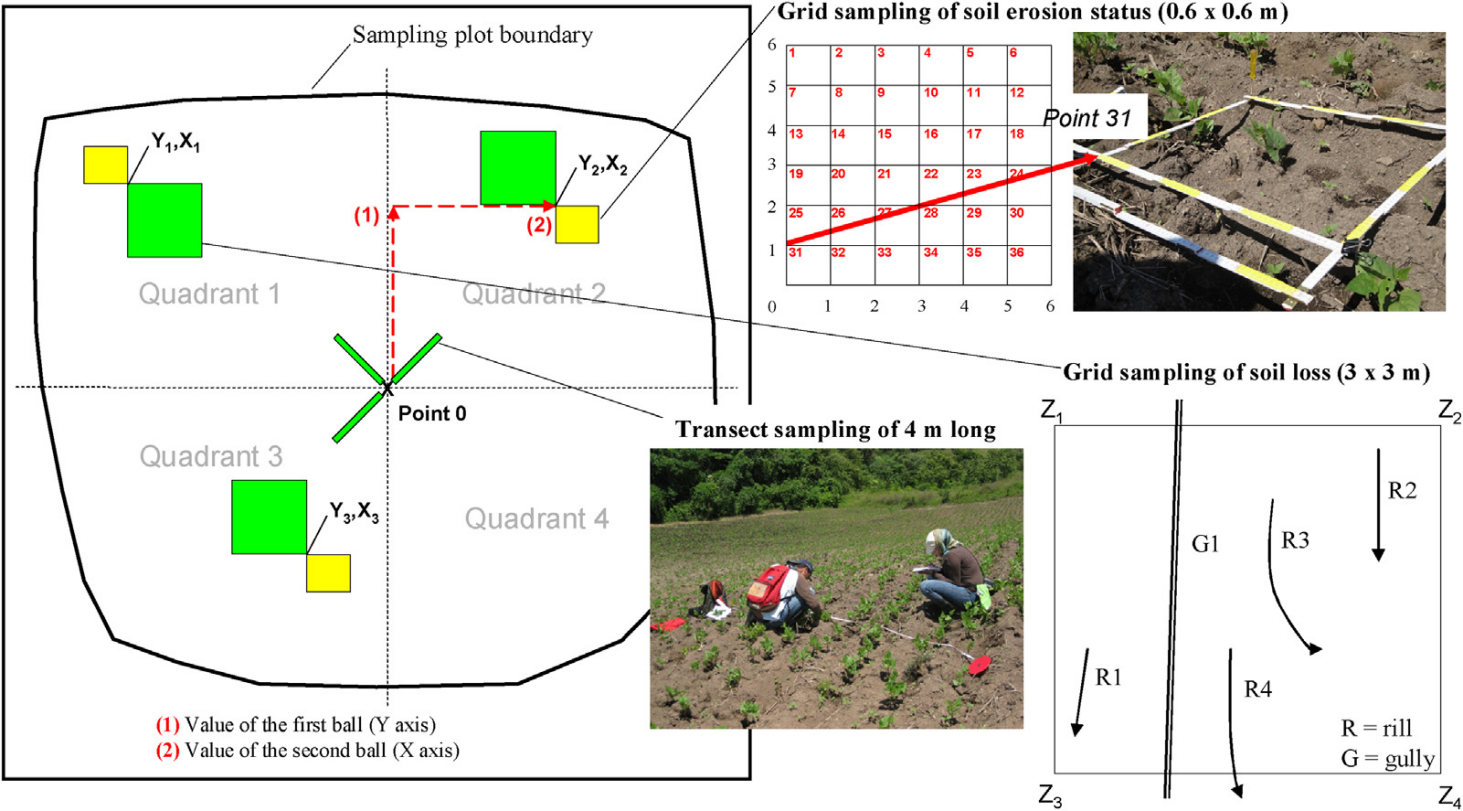
Diagram of sampling plots showing the quadrant selection process and the starting points for the transects and the grid sampling (as described in Blanco and Aguilar [11]). Three sampling grids, measuring 0.6 × 0.6 m are used to analyse the state of soil erosion. At each observation point (36 per grid) the processes (detailed in Table 1) are identified. Another three sampling grids measuring 3 × 3 m are used to analyse the soil loss by erosion. The rills (R) and gullies (G) observed within each grid are located to calculate the volume of soil loss. Diagonal transects measuring 4 m from point 0 are used to analyse the ground cover.

**Fig. 4:**
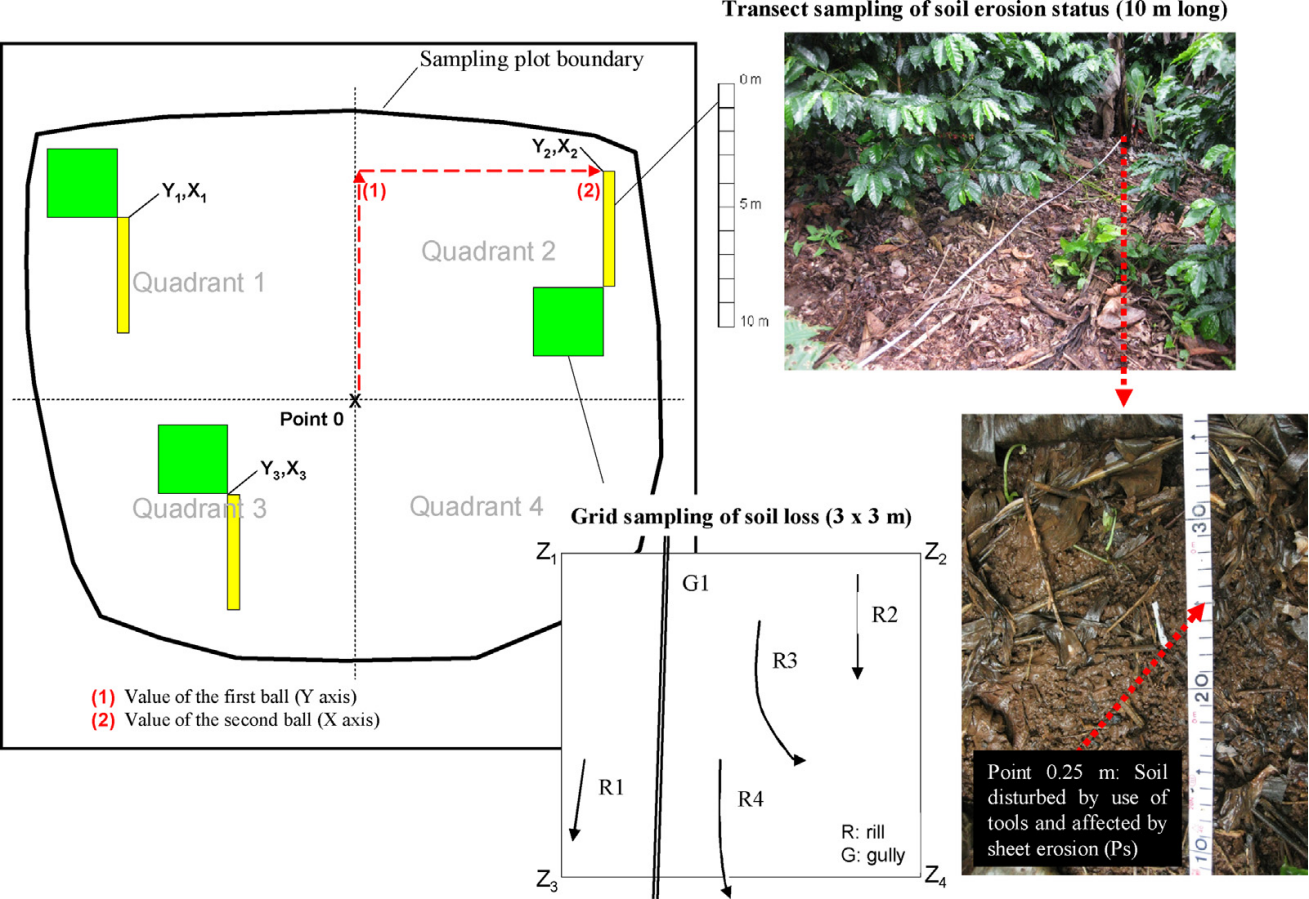
Diagram of sampling plots showing the transect selection process and the starting points for the grid sampling (as described in Blanco and Aguilar [3]). Three sampling transects, each measuring 10 m, are used to analyse the state of soil erosion (as a percentage of the surface area affected) and the ground and aerial cover. At intervals of 0.25 m (40 points per transect) the processes (detailed in Table 1) are identified. Three sampling grids, measuring 3 × 3 m are used to calculate the soil loss by erosion in rills (R) and gullies (G).

In the present study, the sampling method is used to quantitatively evaluate the soil erosion. This method consists of transect or grid sampling and of using grids for soil loss analysis (Fig. 3, Fig. 4) as follows:

(a) The plot is divided into four quadrants.
(b) Three of the quadrants are randomly selected for sampling, by extracting numbered balls from a population n = 4 (4 quadrants).
(c) From point 0 (the convergence of the four quadrants) of the plot, the start point (point Yn, Xn) of the three transects or sampling grids is randomly selected, one in each quadrant, by extracting a numbered ball to determine the distance along each of the axes. Thus, the values of the balls represent the metres to be measured along each axis, from point 0, in order to locate the Yn, Xn point.
(d) Either sampling grids or sampling transects can be used to analyse the state of soil erosion. The choice of method depends on how the land is being used. Sampling grids can be used in studies of pasture farming and of crops such as maize and beans, which are homogeneous and continuous. Sampling transects, on the other hand, can be used in any type of agricultural activity, although they are particularly suitable for heterogeneous and relatively non-continuous uses, that is, where there is a diversity of plant species, such as agroforestry crops. For example, if the sampling grid method were applied to agroforestry systems of coffee cultivation, it might not represent all the different cases that can occur, including: (1) the soil beneath coffee plants; (2) the soil in the inter-rows; (3) the soil beneath trees (which may be of different species). In the transect-sampling method, linear samples, at least 10 m long, are obtained, which makes it unlikely that any of the above-mentioned cases would fail to be represented.
  In the sampling grid method presented, the grids measure 0.6 × 0.6 m. Each side is divided into six sections each measuring 0.1 m, and the sampling point is the convergence of the two axes (Fig. 3). A sample is taken at every 10 cm from point 0, for a total of six points per row (6 points × 6 rows = 36 points per grid). This sampling scheme produces 108 observations per plot (3 grid squares × 36 points, with each point representing 0.9% of the total sample). At each observation point, the processes referred to in Table 1, Table 2 are identified.
  In each plot, the ground cover is quantified (distinguishing between bare soil, weeds, litter layer, stones and others), together with the cover and height of the aerial vegetal strata (distinguishing between herbaceous plants, shrubs and trees). Three sampling programmes are performed, one in each quadrant of those selected in step (b), from diagonal transects 4 m in length, measured from the centre of the plot (point 0) (Fig. 3). The observations are obtained every 10 cm (in total, 40 points per transect). The sampling consists of 120 observations per plot (3 transects × 40 points per transect, with each point representing 0.8% of the total sample).
  In the transect sampling method, the transects used measure 10 m (Fig. 4), and are divided into regular sections of 0.25 m for data collection (in total, 40 points per transect). Thus, at intervals of 0.25 m, over a surface area of approximately 1 cm^2^, the surface process affecting the soil is determined, together with the ground and aerial cover, distinguishing the same classes described above. The sampling was composed of 120 observations per plot (3 transects × 40 points per transect, with each point representing 0.8% of the total sample).
(e) The sampling grids for analysis of soil loss are established from the Yn, Xn points obtained as described above. The grids do not extend beyond the limits of the plot, and their correct location is ensured in accordance with the following order of priority: point Yn, Xn corresponds to the angle Z4–Z3–Z2–Z1 of the grid (Fig. 3, Fig. 4). The grid size is 3 × 3 m. The grid squares need to be as large as possible in order to be representative of the whole plot. Grids measuring 3 × 3 m are normally sufficient to be representative of small plots (<0.3 ha). The rills (R) and gullies (G) observed within the grid are located and listed, to measure their length, width and depth and thus calculate the volume of soil lost. This process is an adaptation and simplification of the ACED (assessment of current erosion damage) method, described by Herweg [9].

Slope gradient is measured in each quadrant using a manual clinometer. The mean value of these measures is taken as the representative slope gradient of the plot. Aerial cover and the height of the vegetal strata are measured using a laser distance meter. During the sampling process, a sample of surface soil (first 10 cm of soil) is taken at point 0 of the sampling plot (Fig. 3, Fig. 4). The samples are air dried, and then the fine fraction of the gravels is separated in a 2 mm mesh sieve. The sieved samples are then analysed in the laboratory. The recommended basic analysis of soil characteristics that can influence erosion includes texture (different fractions), organic matter and calcium carbonate equivalent.

### Erodibility factors

**Fig. 5:**
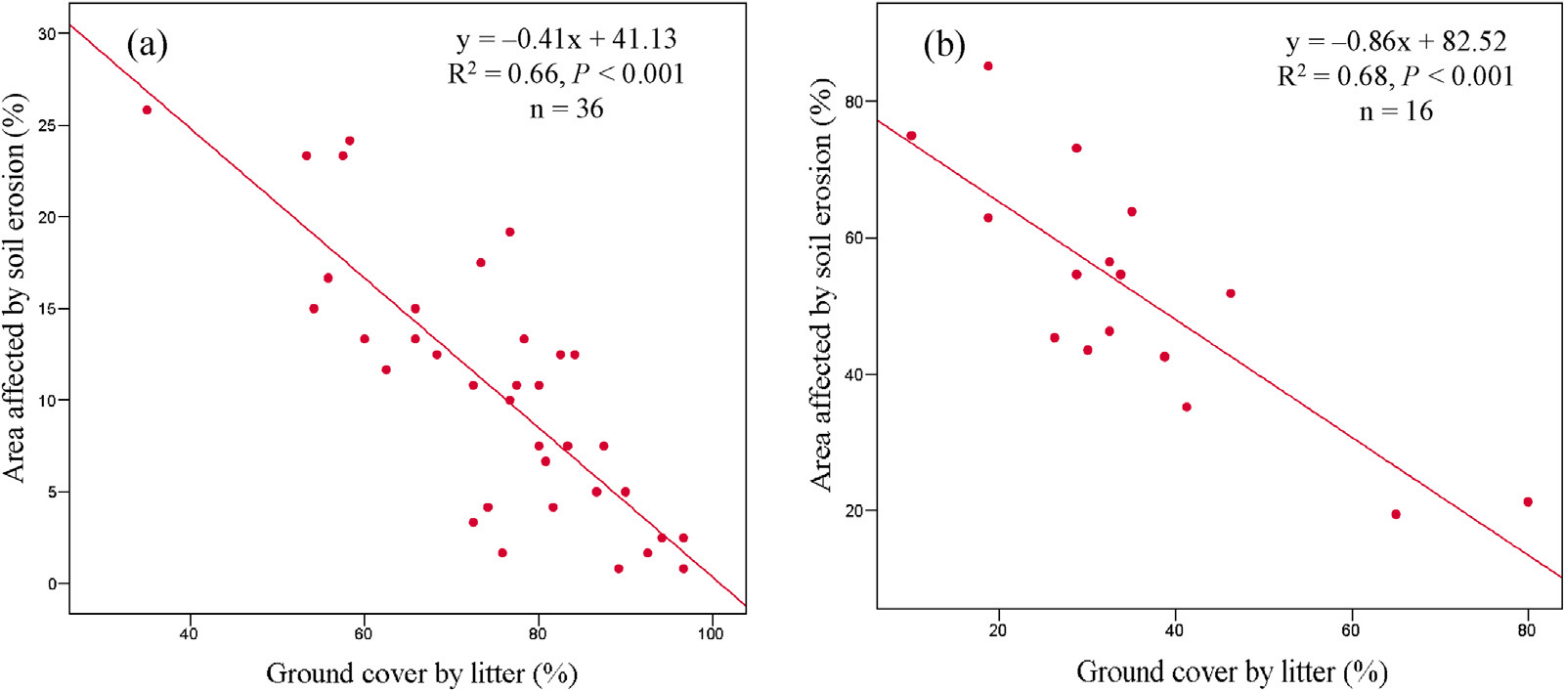
Scatter diagrams and regression curves of the relation between soil affected by erosion and ground cover by litter in an agroforestry system of coffee cultivation (a) (result obtained by Blanco and Aguilar [3]) and in a bean crop (b) (result obtained by Blanco and Aguilar [11]).

A database is created, compiling the results obtained in the erosion sampling and the laboratory analysis of the soil samples, in order to determine the corresponding erodibility factors. These factors are obtained by bivariate correlation and multiple linear regression analysis. The correlation analysis indicates the relationship between the erosive processes and the environmental and management variables. From the regression analysis, applied to the variables presenting a significant correlation with erosion, the erodibility factors (which require a linear relationship between erosion and these variables) can be determined. Fig. 5 shows two examples of results obtained in studies in which this method has been applied.

### Calculating the erosion threshold

Knowledge of the influence of erodibility factors on soil erosion (both environmental and related to agricultural management) can be used to determine erosion thresholds. To establish the erosion threshold, it is necessary to calculate the value (of the erodibility factor) beyond which the reduction in erosion is statistically significant, assuming that water erosion does not increase at a constant rate with respect to erodibility factors. The conditions under which a research study is conducted are of crucial importance in this respect; research conditions in the field (agricultural plots) differ greatly from those in the laboratory and can make it difficult to isolate the influence of each erodibility factor on the overall erosion. For example, Roose and Ndayizigiye [10] showed that erosion did not increase linearly with the slope gradient, due to the existence of a stonier soil and less crusting on the steeper slopes, which reduced the degree of erosion. That is, the erosion trend observed with respect to a particular factor may be influenced by other factors, the interference from which is difficult to isolate, given the working conditions in the field plots. Nevertheless, these are the actual conditions that must subsequently be addressed when research outcomes are used as the basis for management recommendations. From this standpoint, it seems most appropriate to conduct the study under the same conditions as those to which the results will later be transferred.

The erosion threshold, in relation to erodibility factors, is obtained by means of two types of variance analysis. When the database analysed has n < 30 and there is a risk of non-normal distribution of values, non-parametric statistical techniques are recommended, such as the Kruskal-Wallis test for several independent variables and the Mann–Whitney test for two independent variables. In the present study, therefore, a general analysis is first performed in which the results of erosion are distributed at different intervals, using the soil erodibility factors as the grouping variable. Two criteria are applied for this purpose: (a) the distribution should present regular intervals, that is, representing a constant rate of growth (for example, at intervals of 10: 10–20, 20–30; or of 20: 10–30, 30–50), because the variance is sensitive to the range if the results present a high degree of dispersion; (b) the distribution should present a minimum size (n) of 3 cases in each interval. The second analysis concerns the variance between the erosion and the erodibility factors previously found to be significant, and is performed by examining pairs of consecutive intervals. The erosion threshold is established at the boundary between the two intervals of the pairs analysis for which the highest level of statistical significance is obtained. See Blanco and Aguilar [3,11] for applications of this method.

### Verifying the erosion threshold

**Fig. 6:**
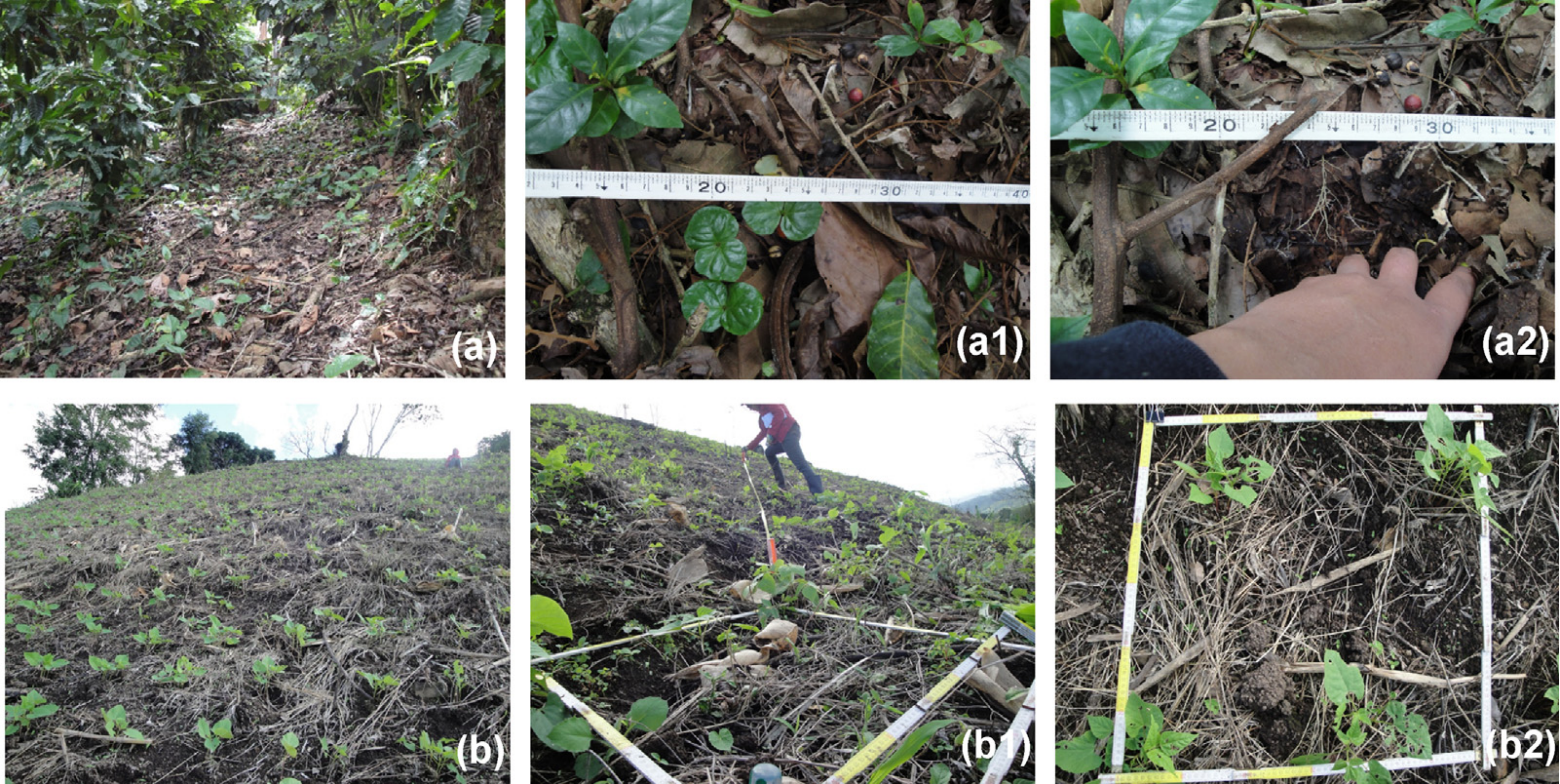
No erosion and slight intensity of soil erosion by water (classes 0 and 1 of the Table 3) in coffee (a) and bean (b) crop cultivations in relation to the litter layer as erodibility factor (adapted from Blanco and Aguilar [3,11]): (a) Plot with 87% litter layer. Non-eroded soil beneath a cover of vegetal residue (a1). Under this cover, decomposing plant remains are visible, which indicates that the soil is not affected by erosion (a2) (class 0 of the Table 3). (b) Plot with 65% litter layer. Splash and sheet erosion effects are visible at some points, but only in areas not covered by plant residues (b1, b2) (class 1 of Table 3).

**Fig. 7:**
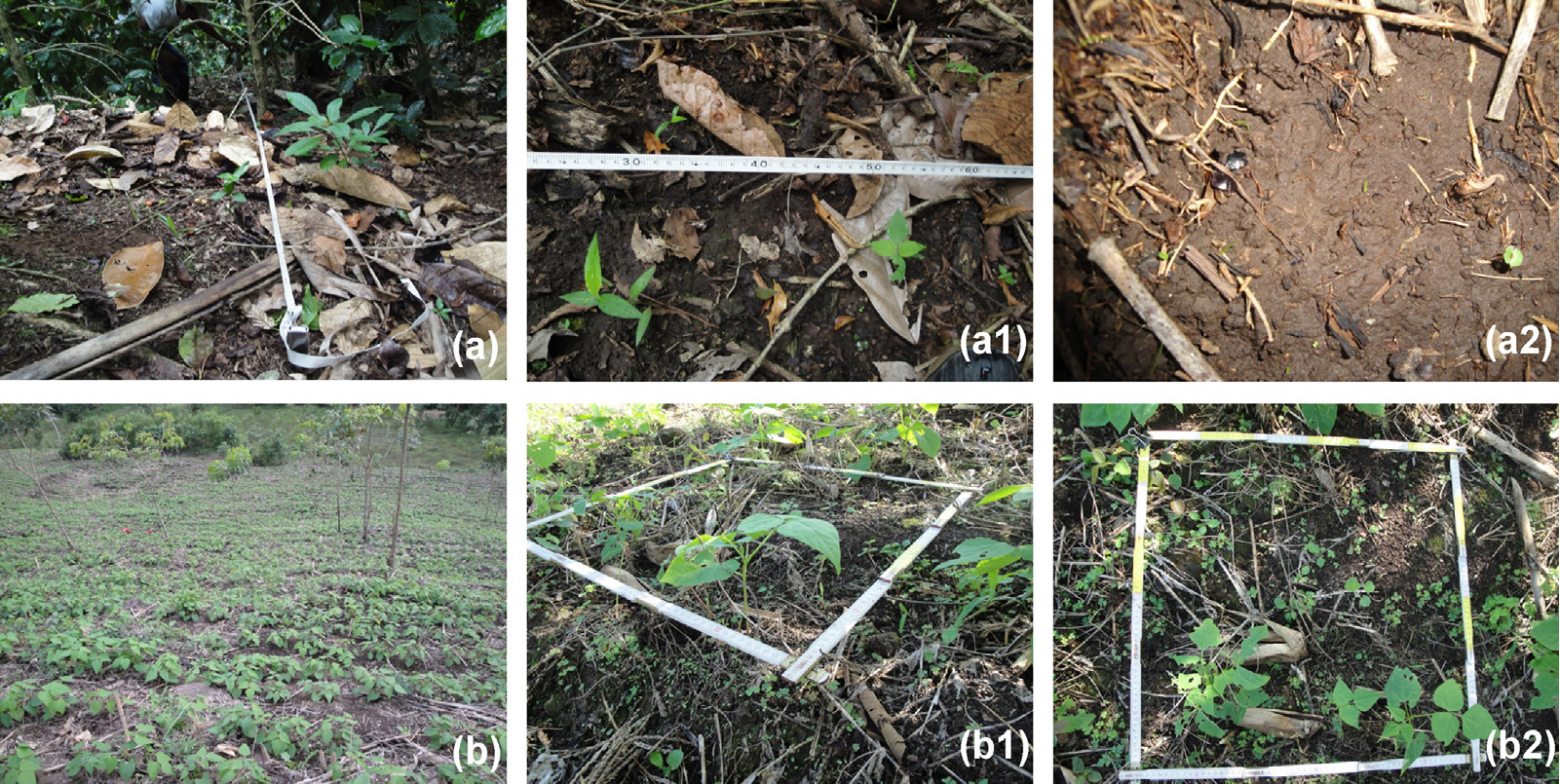
Moderate intensity of soil erosion by water (class 2 of Table 3) in coffee (a) and bean (b) crop cultivations in relation to the litter layer as erodibility factor (adapted from Blanco and Aguilar [3,11]): (a) Plot with 58% litter layer. Splash and sheet erosion effects are visible but most of the surface area is unaffected (a1). Detail of sheet erosion (a2). (b) Plot with 46% litter layer. Splash and sheet erosion effects are visible but not predominant (b2).

**Fig. 8:**
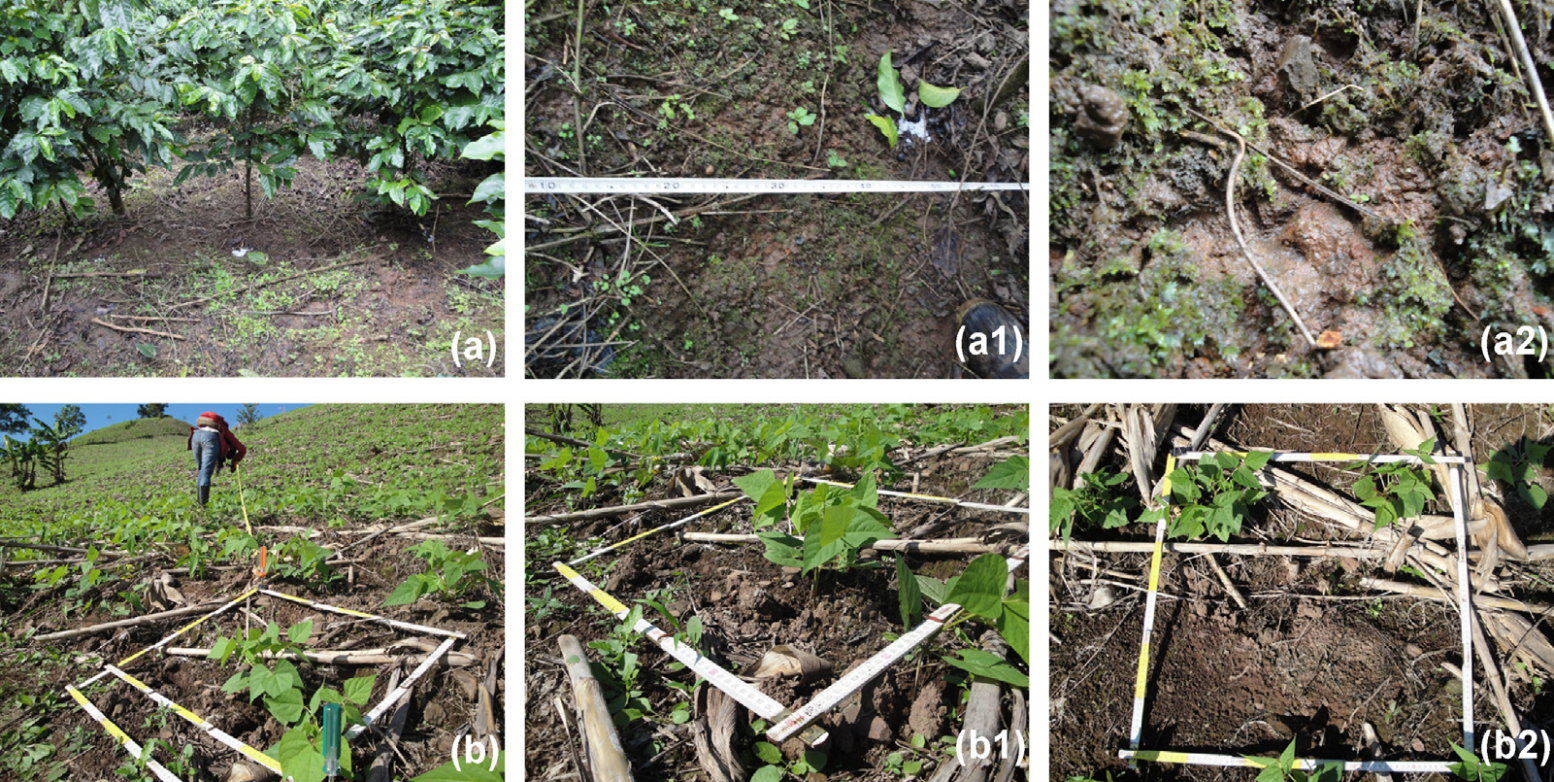
Severe intensity of soil erosion by water (class 3 of Table 3) in coffee (a) and bean (b) crop cultivations in relation to the litter layer as erodibility factor (adapted from Blanco and Aguilar [3,11]): (a) Plot with 35% litter layer. Splash and sheet erosion effects are clearly visible and affect the entire plot (a1). Detail of splash and sheet erosion (a2). (b) Plot with 35% litter layer. Splash and sheet erosion effects are very visible over most of the surface area (b2).

It is important to recognise that reducing erosion in statistical terms is not synonymous with the effective control of erosion, because a “statistical” reduction may take place at high absolute values of erosion. To assess whether the erosion threshold used corresponds to effective erosion control, a verification method is applied, based on the use of visual indicators to assess the intensity of erosion by means of a qualitative classification (Table 3). It has been established that an erosion threshold, determined from a statistical standpoint, reflects the effective control of erosion when a plot with the specified characteristics presents an erosion intensity of category 0 or category 1 (Fig. 6); in contrast, it is not effective when the category is 2 (Fig. 7) or 3 (Fig. 8). In the latter cases, the erosion threshold cannot be used for the purpose for which it was designed.

**Table 3:** Qualitative classification of the evaluation of the intensity of soil erosion by water (Blanco and Aguilar [3]).

Score (cod)	Intensity of erosion	Explanation
0	None/very slight	The soil is not affected or is only slightly affected by erosion. In the latter case, it is not immediately apparent. Evidence of erosion is occasional and only present in certain specific places, such as at the base of trees, bushes or rocks.
1	Slight	The soil is somewhat affected by erosion, and this is clearly visible throughout the plot. The evidence of erosion is more widespread and also more apparent than in the previous case, but is only very clearly concentrated in very specific locations.
2	Moderate	The soil is clearly affected by a moderate degree of erosion. Evidence of erosion is widespread and may be concentrated at any point on the plot, but the majority of the surface area remains unaffected by erosion.
3	Severe	The soil is strongly affected by erosion. The majority of the surface area is affected by erosion (in case of doubt, the plot is classified as Class 2).
