# Supplementary material for: An erosion control and soil conservation method for agrarian uses based on determining the erosion threshold
Source: MethodsX. 2018 Jul 9;5:761–72. doi: 10.1016/j.mex.2018.07.007 (PMC6090081; doi:10.1016/j.mex.2018.07.007)
Supplement: Supplementary file 1 [file mmc1.doc]

**Appendix 1:**  **Reconnaissance of the study area**

Before applying the method described, it may be necessary to conduct a reconnaissance of the study area, which would consist in performing a survey of the area, farm by farm, in order to diagnose the erosive situations currently affecting the soil as the result of agricultural activity. This reconnaissance also facilitates diagnosis of the main factors involved in the erosion. The aim of this reconnaissance activity is to establish the research goals and to select representative plots in which to conduct the study. These plots must be homogeneous in terms of the physical and human factors that may influence erosion processes.

The field survey consists of an informal interview with the farmer followed by a tour of the different plots that make up the farm. The aim of these interviews is to obtain information with which to analyse the use and management of the land on each farm, as these considerations can have a major influence on erosion, as indicated below. Tables 1 to 3 provide examples of interview scripts designed for studies of annual herbaceous crops (maize and beans), shrubs and trees (coffee and cacao) and livestock, in the mountains of Central America.

**Table 1**

Interview script on management practices for annual herbaceous crops (maize and beans).

| **- Surface area:**  **- History of land use:** 🞎 The same as at present (years: ), 🞎 Other uses:  **- Crop management, tools used and timetable:** (1) Pre-seeding management; (2) Seeding; (3) Post-seeding management; (4) Harvesting.   | Questions included in analysis of management: 🞎 Do you allow cattle to enter the plot to graze the stubble? 🞎 Do you burn off the stubble? 🞎 Ploughing (direction of ploughing: ), 🞎 Tools for weeding and seeding: digging stick, machete (type: ), hoe, pickaxe, 🞎 Tools for seeding (type: ). | | --- |   **- Erosion control practices and measures:**  **- Yield:** |
| --- | --- |

**Table 2**

Interview script on management practices for tree and shrub crops (coffee and cacao).

| **- Surface area:**  **- History of land use:** 🞎 The same as at present (years: ), 🞎 Other uses:  **- Crop management, tools used and timetable:** (1) Fertilising; (2) Weeding; (3) Harvesting.   | Tools used for weeding: 🞎 Machete (type: ), 🞎 Others: | | --- |   **- Separation between plants (cm):**  **- Shade cover (identify species) and height of branches (distance to the ground) (cm):**  **- Erosion control practices and measures:**  **- Yield:** |
| --- | --- |

**Table 3**

Interview script on management practices for pastures for extensive cattle rearing.

| **- Surface area:**  **- History of land use:** 🞎 The same as at present (years: ), 🞎 Other uses:  **- Type and size of cattle herd:**  **- Size of pastures:**  **- Type of pasture:** 🞎 Wild species:  🞎 Enhanced species:  **- Grazing system:**  a) Grazing and resting periods for each pasture:  b) Number of animals that enter the pastures in each phase of the grazing cycle:  **- Erosion control practices and measures:**  **- Yield:** |
| --- |

The field trips around the plots form part of a preliminary, rapid study of the environmental characteristics. The methods applied are direct and straightforward, to ensure that the data acquisition process is as fast and effective as possible. The first consideration made is of certain characteristics of particular interest due to their influence on erosion (Table 4). The slope of the ground is determined using a clinometer and the lithology is identified. The texture is identified indirectly by asking the farmer if the plot becomes muddy when it rains. This inquiry reveals whether the topsoil has a high content of clays. This aspect is then verified by applying the finger assessment procedure described by McRae [1]. The vegetal cover is qualitatively evaluated by observation of the soil surface with respect to four parameters, as described in Table 4. Secondly, a preliminary assessment is made of the state of soil degradation, according to visual indicators (Fig. 1). The aim of this examination is to identify the presence in the soil of mass movements, erosion and impacts by grazing. The magnitude of the erosion is assessed by measuring the length, width and depth of the rills and gullies. In all cases, the impacts are evaluated qualitatively on a three-point scale: occasional 🞎 (indicated by 🗷), frequent (+) and very frequent (++) (in the latter two cases, this is indicated by underlining the symbol).

To perform such a reconnaissance in other study areas, of course, the interview script and the assessment scheme of impacts must be adapted to the characteristics of the area in question.

**Table 4**

Environmental characteristics of the plot.

| **- Slope:**  **- Lithology:**  **- Texture:** Does mud form when it rains?: Finger assessment procedure:  **- Vegetal cover:** 🞎 None 🞎 Poor (many bare patches), 🞎 Good (some bare patches), 🞎 Complete. |
| --- |

**Fig. 1.** Qualitative assessment of types of soil degradation frequently observed in agriculture and in cattle farming in mountainous areas of Central America.

**References**

[1] S.G. McRae, Practical pedology: Studying soils in the field. Ed. John Wiley. New York, US, 1988, 253 p.
